# Late Preterm Infants' Social Competence, Motor Development, and Cognition

**DOI:** 10.3389/fpsyt.2019.00069

**Published:** 2019-02-20

**Authors:** Jia You, Hong-juan Yang, Mei-chen Hao, Jing-jing Zheng

**Affiliations:** Early Child Development Center, Xi'an Maternal and Child Health Care Hospital, Xi'an, China

**Keywords:** late preterm infant, social competence, cognition, motor disorder, risk factor

## Abstract

The aim of this study was to compare the social competence, motor development, and cognition of late preterm infants (LPIs) with full-term infants. Several studies in the recent past indicated that LPIs are at high risk of social development problems. We compared the development of motor skills, cognition, and social competency of LPIs with full-term infants at between 2 and 2.5 years old. The Chinese versions of the Gesell Development Diagnosis scale and the Normal Development of Social Skills from Infants to Junior High School Children scale were used for the assessment. LPIs were not more socially competent than their full-term counterparts. Each skill—namely, adaptability, gross motor, fine motor, language, and personal-social responses—was separately associated with the total level of social skills. It was found that gross motor skills had a positive correlation with the self-help and locomotive abilities, and fine motor skills had a positive association with locomotion abilities. LPIs had risk factors due to their delayed social skills in areas including motor disorders and physiological and perinatal factors. LPIs under three were at a higher risk of impairment in social competency. Therefore, it is recommended that they be monitored regularly to identify the development of social and cognitive disorders at an early stage.

## Key Concepts

Late preterm infants: The 2005 National Institutes of Health workshop recommended that infants born at 34 0/7 through 36 6/7 weeks' gestation after the onset of the mother's last menstrual period be referred to as “late preterm.” Used in lines 12, 14, 17, etc.

Social competence: This refers to the ability to interact and communicate with others by predicting and understanding others' behaviors, and to engage, explore, handle frustration, show self-control, and so on. Used in lines 14,16, 20, etc.

Cerebral palsy: Cerebral palsy describes a group of permanent disorders affecting the development of movement and posture, causing activity limitation, that are attributed to non-progressive disturbances that occurred in the developing fetal or infant brain. The motor disorders of cerebral palsy are often accompanied by disturbances of sensation, perception, cognition, communication, and behavior by epilepsy and by secondary musculoskeletal problems (Modified after (1)). Used in line 36.

Cognition delay: A full-scale score on the Gesell Development Diagnosis Scale lower than 75 is defined as cognition delay. Used in lines 34 and 222.

Motor disorders: Both motor delay and cerebral palsy are classified as motor disorders. Used in lines 131,174, 196, etc.

## Introduction

In the field of medicine, the neurodevelopment of preterm infants has always caused concern. In the last 10 years, studies were conducted on very preterm infants and extremely preterm infants. Those infants were reportedly at high risk of developing neurological disorders such as cerebral palsy (2); cognitive delay (3); and specific behavioral problems like attention-deficit/hyperactivity disorder (4), autism (5), and executive dysfunction (6, 7). Preterm infants at a gestational age (GA) of <32 weeks were prone to a higher risk of disability (8).

A preterm birth with a GA of 34 weeks 0 days to 36 weeks 6 days is called a late preterm birth; 70% of preterm births fall into this gestation period (9). Until a few years ago, late preterm birth was considered of no importance in the regular monitoring of babies' health, neurodevelopment, and social development. In fact, late preterm infants (LPIs) were considered normal and only preterm infants with a GA of <32 weeks were considered to be high risk. Also, LPIs had nearly normal birth weights and did not show any clinical problems (10). However, recent studies on LPIs confirmed that they are also at high risk of mortality as well as of multiple morbidities when compared to full-term infants (FTIs). LPIs were reported to suffer from respiratory problems, apnea, jaundice, feeding difficulties, infections, and other evident life-threatening events (11, 12). Above all, it was reported that LPIs are at high risk of impaired neurodevelopment. Compared to FTIs, LPIs have a higher prevalence of motor disorders and psychological and behavioral problems. Children with a GA of 36 weeks require special educational services support. Also, they have clinical attention challenges. This continues to be a problem for LPIs into adulthood and they make slow progress in academics and attain lower social status than FTIs (13). These concerns emphasize the need to discuss the social competency of LPIs.

Socialization is a fundamental social development skill that is required for the psychological development of a child. The linear progression of this skill from childhood on helps them to be independent and social when they grow into adults (14). Social development or social competence spans social adaptation, social emotion, social cognition (15), and social interaction, as shown in many studies (16). Social competence has numerous meanings, but it is basically the skill acquired by the individual to get along with others. Social cognition refers to the cognitive processes that include interaction with others to understand their intentions, feelings, emotions, and behaviors. Social skills include a wide range of abilities that emerge from the appropriate execution of social cognition. Social functioning refers to the social behaviors that are performed every day and are maintained in social contexts (17). Social emotion is the ability to talk and interact with others and thereby be able to predict and understand their behaviors. Also, it is one's ability to develop relationships with others by controlling and handling frustration (18). Social cognition, emotion, competence, development, adaptation, etc., all have interrelated meanings, but social cognition is regarded as the key variable in social competence (17). A lack of social competence in children will lead to poor academic achievement and affect mental stability (19). Children will also have limited social, occupational, and family functioning abilities when they grow up (20).

Dueker et al. conducted a study to predict the development of the communication, personal-social, and problem-solving abilities of LPIs in different time durations till they reached the age of 2.5 years. They confirmed that there was a delay in the development of the above-mentioned skills (21). Other studies confirmed that LPIs had worse overall social and emotional competence when compared to FTIs (22, 23). However, other studies indicated that LPIs were the same as their FTI counterparts, with no serious issues (24).

However, research has seldom explored the association between cognition/motor abilities and social competence. Also, many LPIs have poor social skills and delayed motor/cognitive skills in clinical practice. This leads to the need for a study to identify and treat the early signs of abnormal social skills in LPIs to help parents develop a positive parenting pattern.

Throughout the study, we consider LPIs as being at high risk of delayed social competence, and as developing social skills later in life than FTIs, who are traditionally considered “low risk.” Additionally, the cognition/motor abilities of LPIs are also correlated with social competence. The aim of this study is to compare and correlate the social competence, cognition, and motor development of LPIs with FTIs. Also, the risk factors associated with delayed social competence are studied.

## Materials and Methods

### Participants

This retrospective study included 112 LPIs born between October 2013 and October 2015 with a GA between 34^+0^w and 36^+6^w. They were considered healthy and had no serious clinical issues post birth. The study also included 179 healthy FTIs of the same age in the same geographic area who had no perinatal risk factors as controls.

### Procedure

The children had regular health check-ups from birth until 3 years of age in the Early Child Development Center of the hospital. Until the child was 6 months old, the checkup interval was once a month, and then once every 2 months after that. The physical examination included the measurement of weight, height, and head circumference. Laboratory tests for excluding clinical diseases were done. A neurodevelopment screening to examine neuro-reflects, postures, motor function, and social behaviors was carried out. Suggestions to parents were provided based on the examination results. Every mother of a child aged 24–30 months was asked to complete questionnaires on the GA, birth weight, mode of delivery, perinatal risk factors, maternal education status, financial status, etc. Later on, the infants went through physical and neurological assessments. The birth date and birthplace of both LPIs and FTIs were in the same time period and locality. We obtained approval for this study from the Ethics Committee of the hospital. Each mother signed a written informed consent form before filling out the questionnaire.

#### Normal Development of Social Skills From Infants to Junior High School Children (S-M)-Chinese Version

This is a 132-item scale with 6 subdomains, as explained below.

1. **Self-help** refers to the ability to brush teeth, take a bath, wear clothing, eat, and keep their surroundings clean.
2. **Locomotion** includes the ability to walk, climb stairs, play outdoors, cross roads, visit friends, go to school alone, understand, and obey traffic signs even in unfamiliar places, etc.
3. **Occupation** includes the abilities to clutch; crawl; pour milk; prepare and clean tableware; use tools such as glue, screwdrivers, scissors, and gas burners; cook; heat water; repair furniture; etc.
4. **Communication** includes the abilities to say their name, respond to calls, understand simple instructions, recall memories, express their views to people, read and understand newspapers, leave notes, write letters, refer to a dictionary, etc.
5. **Socialization** includes the abilities to take part in group events, communicate with peers, participate in activities in or outside of school, represent his/her class, organize trips, etc.
6. **Self-direction** includes the abilities to think and execute things. This includes planning, managing time, studying, sleeping on time, and in short handling oneself without others' support.

The 132-item scale is arranged in a sequence of development across each subdomain. Rating is done on a two-point scale (Yes = 1, No = 2) for each item. Rating is terminated when there are 10 consecutive “Nos.” Computers are used to standardize both the subdomain scores and the overall scores. Based on the final score, the S-M skills are classified as extremely severely abnormal (score < 5), severely abnormal (score = 6), moderately abnormal (score = 7), mildly abnormal (score = 8), borderline (score = 9), normal (score = 10), above normal (score = 11), and excellent (score = 12).

#### Motor Delay and Cerebral Palsy

Neurological examinations detected cerebral palsy in some infants and standard criteria were used to diagnose the same (25). Motor ability falling 3 months behind age-related milestones is called motor delay. Also, a development quotient (DQ) <75 for gross motor skills on the Gesell Development Diagnosis Scale (GDDS) signifies motor delay. Infants were confirmed to have a motor disorder based on the development of both motor delay and cerebral palsy.

#### Chinese Version of Gesell Development Diagnosis Scale (26)

The Chinese version of the Gesell Development Diagnosis Scale (GDDS) has five domains: adaptability, gross motor, fine motor, language, and personal-social responses. The full-scale DQs for each domain were calculated for all participants. Based on the full-scale DQ results, the development of infants was classified as normal (DQ ≥ 85), deficient (DQ <7 5), or borderline (75 ≤ DQ < 85). A DQ in any single domain falling below 75 was also considered deficient within that domain.

### Data Analysis

Comparisons between two independent samples and the analysis of potential risk factors were carried out in a case-control study.

First, the sample size was fixed using the formula N1 = N2 = 2[(t½α+t½β)σ]^2^/δ^2^. “σ” means the estimation of the standard deviation between two populations. “δ” is the difference between two means. “t½α” is the t value when the level of a test “α” is 0.05 and “t½β” is the *t*-value when the type II error is 0.1.

The minimum of the sample was obtained using the scores in the subdomain of “self-help.” This study included a minimum of 50 FTIs and LPIs each. Besides, 65–130 children needed to be included in this study, as the sample size had to be 5–10 times larger than the influential factors.

In our study, the numbers of LPIs and FTIs were bigger than the computing results. Data analyses were performed with the Statistical Package for the Social Sciences (SPSS) version 18.0 for Windows. The descriptive data were presented as [[Inline Image]] ± SD. Chi-square tests and Fisher's exact probability were used to compare the percentages. A Student's *t-*test for two independent samples was performed to compare the means of the groups. Person's correlation coefficient was used for correlation analysis. A multivariate linear regression was performed to analyze the risk factors of S-M including birth weight, GA, perinatal high-risk factors, and dyskinesia. *P*-values of <0.05 were considered statistically significant. The Bonferroni method was used to adjust the alpha level while the differences between LPIs and FTIs in the domain scale were compared.

## Results

### Characteristics of the Study Population

A total of 61 and 85 boys from the LPI and FTI groups, respectively, were included in the study. Infants' characteristics including GA, mean corrected age, average birth weight, mode of delivery, perinatal risk factors, maternal education, and financial status were collected and compared. The characteristics are shown in Table 1. The average GA showed a significant difference between LPIs and FTIs (GA: 35.02 ± 1.95 vs. 39.15 ± 1.50 weeks). There were no significant differences between the two groups in terms of age/corrected age (*t* = −1.818, *p* = 0.070), sex (x^2^ = 1.342, *p* = 0.247), birth weight (x^2^ = −0.096, *p* = 0.321), mode of delivery (x^2^ = 1.930, *p* = 0.165), maternal education (x^2^ = 1.547, *p* = 0.214), and financial status (x^2^ = 0.799, *p* = 0.671). Sixty-nine LPIs had at least one perinatal risk factor, of which 14 had more than 1 risk factor. The distribution of the perinatal risk factors of LPIs is shown in Table 2.

**Table 1:** Characteristics of LPIs and FTIs.

	**LPIs *n* (112)**	**FTIs *n* (179)**	***x*^**2**^ or *t*-value**	***p*-value**
Male, *n* (%)	61 (54.5)	85 (47.5)	1.342	0.247
Age/corrected age	26.77 (2.37)	26.24 (2.38)	−1.818	0.070
Gestational age (weeks)-mean (SD)	35.02 (1.95)	39.15 (1.50)	−22.76	0.000
Birth weight (grams)-mean (SD)	2,774 (4,690)	3,390 (510)	−0.996	0.321
Delivery mode, *n* (%)	Not available	Not available	1.930	0.165
Natural labor	39 (34.8)	77 (43.0)		
Cesarean section	73 (65.2)	102 (57.0)		
Perinatal risk factors, *n* (%)	75 (67.0)	0 (0)		0.000
Maternal education, *n* (%)			1.547	0.214
Completion of university	69 (61.6)	114 (63.7)		
Completion of secondary school	43 (38.4)	65 (36.3)		
Family income, *n* (%)	Not available	Not available	0.799	0.671
High	39/102 (38.2)	59 (33.0)		
Medium	48/102 (47.1)	91 (50.8)		
Low	15/102 (14.7)	29 (16.2)		

**Table 2:** Perinatal risk factors in LPIs.

**Risk factors**	**Twin birth**	**Pregnancy-induced hypertension**	**Placenta previa**	**Incipient abortion**	**Cholestasis**	**Oligohydramnios**	**Gestational diabetes**	**Intracranial hemorrhage**	**Cord round the neck**	**Amniotic membrane infection**	**Intrauterine distress**
LPIs(n)	36	21	10	7	5	2	2	2	1	1	1

### Social Competence of the LPIs and FTIs

**Figure 1:**
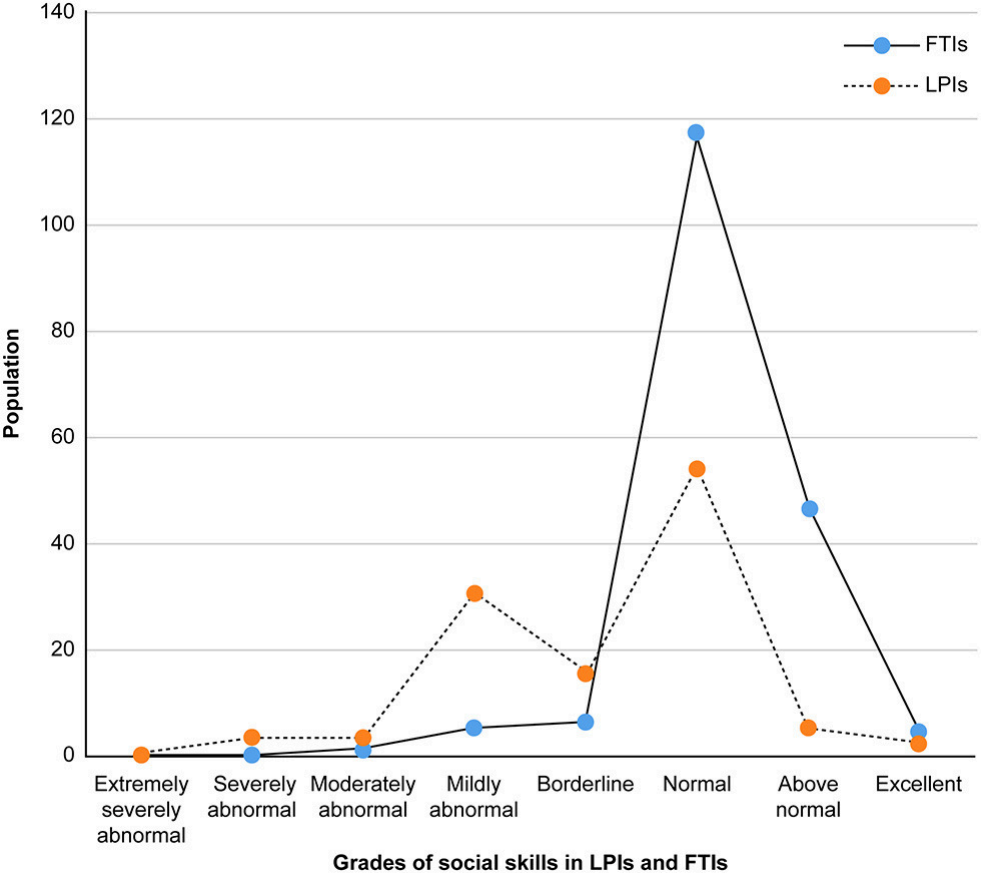
Distribution of grades of social skills in LPIs and FTIs.

Compared with FTIs, LPIs presented worse social competence. The majority of LPIs had abnormal S-M grades, as shown in Figure 1. FTIs had predominantly normal to excellent S-M grades. The total S-M score for LPIs was significantly lower than that for FTIs. LPIs showed inferior performance when compared to FTIs in the locomotion and self-help subdomains. The remaining subdomains were more likely to be the same between the two groups. Still, the mean scores of the occupation, communication, socialization, and self-regulation subdomains were lower for LPIs than FTIs (Table 3).

**Table 3:** Comparison of scores in S-M between LPIs and FTIs (^*x*^ ± s).

**Item**	**LPIs *n* (112)**	**FTIs *n* (179)**	***t*-value**	***p*-value**
S-M	9.06 ± 1.90	10.03 ± 0.97	−2.864	0.005
Self-help	5.09 ± 1.73	8.39 ± 2.86	−7.321	0.000
Locomotion	3.68 ± 1.49	4.53 ± 1.40	−2.826	0.006
Occupation	4.00 ± 1.21	4.31 ± 1.43	−1.15	0.253
Communication	5.18 ± 1.56	5.75 ± 1.52	−1.80	0.075
Socialization	6.41 ± 1.81	6.83 ± 1.65	−1.12	0.246
Self-regulation	3.09 ± 1.93	3.56 ± 1.65	−1.23	0.22

### Motor Abilities and Cognition in the Two Groups

Two LPIs were diagnosed with spastic diplegia. The classic clinical signs such as motor abilities falling behind by a minimum of 3 months of the age-corresponding milestone, hypermyotonia, posture, and abnormal reflexes were present. There were abnormalities indicated in their GDDS subdomain scores. Nine LPIs had a motor delay and inferior motor abilities and no abnormal gestures and reflexes. Three out of the 9 had mild low or high muscle tone. LPIs had an overall rate of motor disorders of 9.82% (11/112). None of the FTIs had cerebral palsy although one child exhibited a simple motor delay. This resulted in a 0.56% rate of motor disorders in this group (1/179). The two groups were significantly different in terms of motor disorders (Fisher's exact probability *p* < 0.01). The GDDS assessment (Table 4) also indicated that LPIs' showed inferior performance and lower mean scores in the subdomains of adaptive behavior, gross motor, fine motor, language, and personal-social responses. Also, there were greater discrepancies in each developmental field when LPIs and FTIs were compared (all *p* < 0.01).

**Table 4:** Comparison of GDDS scores between LPIs and FTIs (^*x*^ ± s).

**Items**	**LPIs *n* = 112**	**FTIs *n* = 179**	***t-*value**	***p-*value**
Adaptive behavior	77.52 ± 13.92	93.72 ± 10.34	−11.48	0.000
Gross motor	84.02 ± 14.18	96.31 ± 10.23	−8.19	0.000
Fine motor	76.52 ± 9.44	93.18 ± 11.69	−10.11	0.000
Language	75.39 ± 14.60	94.97 ± 14.52	−8.93	0.000
Personal-social responses	77.44 ± 13.21	96.70 ± 11.56	−10.64	0.000

### Correlations of Abilities in GDDS With Social Skills in LPIs

A Pearson's correlation analysis showed that abilities in GDDS were moderately and highly correlated with the total level of S-M (all *p* < 0.001). Gross motor skills and fine motor skills were positively correlated with the self-help, locomotive, and locomotion skills. The vales were as follows: *r*-value = 0.264 and 0.355; both *p* < 0.001, *r*-value = 0.332; *p* < 0.05, respectively. Adaptive behavior, language, and personal-social skills in GDDS were not positively correlated with the subdomains of S-M. Gross motor skills were not associated with occupation, communication, socialization, and self-regulation skills, and fine motor skills were not associated with self-help, occupation, communication, socialization, and self-regulation skills (all *p* > 0.05) (Table 5).

**Table 5:** Association of domain skills between the GDDS and S-M in LPIs.

		**S-M**	**Self-help**	**Locomotion**	**Occupation**	**Communication**	**Socialization**	**Self-regulation**
Adaptive behavior	Pearson's correlation	0.829	0.069	0.000	−0.088	−0.075	−0.177	−0.199
	Significance(bilateral)	0.000	0.583	0.997	0.486	0.552	0.097	0.270
Gross motor	Pearson's correlation	0.656	0.264	0.355	0.149	0.038	−0.050	0.062
	Significance(bilateral)	0.000	0.041	0.005	0.268	0.654	0.513	0.576
Fine motor	Pearson's correlation	0.646	0.145	0.332	0.044	0.068	−0.025	−0.075
	Significance(bilateral)	0.000	0.360	0.032	0.786	0.676	0.820	0.502
Language	Pearson's correlation	0.635	−0.012	−0.061	−0.048	0.085	−0.087	−0.002
	Significance(bilateral)	0.000	0.936	0.685	0.752	0.576	0.485	0.984
Personal-social responses	Pearson's correlation	0.638	0.093	−0.158	−0.180	−0.068	−0.010	0.033
	Significance(bilateral)	0.000	0.540	0.294	0.231	0.655	0.934	0.792

### Risk Factors of Delayed Social Development in LPIs

The effects of different biological factors such as GA, birth weight, motor disorders, and perinatal factors of LPIs were assessed. The perinatal factors included twin birth, intrauterine distress, hyperbilirubinemia, pregnancy-induced hypertension (PIH), gestational diabetes, placenta previa, umbilical cord around the neck, oligohydramnios, amniotic membrane infection, and intracranial hemorrhage. Two modes were created to identify the majority of the predictive factors. GA, birth weight, and perinatal risk factors were included in Mode 1. Mode 2 had motor disorders and the Mode 1 factors. All of these factors are clinically important. They are documented as common predictors of adverse outcomes of preterm birth and neonatal morbidities. A multiple linear regression analysis showed the following:

- PIH (Mode 1, Mode 2) and motor disorders (Mode 2) affected the adaptability domain of GDDS (B = 18.83, 15.66,−18.49; *p* = 0.011, 0.020, 0.000; 95% CI: 4.461 to 33.196, 2.518 to 28.797,−26.984 to−9.988, respectively).
- Placenta previa (Mode 1) and motor disorders (Mode 2) were predictors of personal-social behavior (B = −10.35, −15.61; *p* = 0.043, 0.001; 95%CI: 0.523–29.956, −24.586 to −6.631, respectively).
- PIH (Mode 1, Mode 2) and GA, oligohydramnios, and motor disorders (Mode 2) affected the overall ability domain of S-M (B = 1.46, 0.199, 1.160, −1.62, −5.585; *p* = 0.012, 0.050, 0.020, 0.021, 0.000; 95% CI: 0.334–2.593, −0.004 to 0.403, 0.185 to 2.135, −2.984 to −0.252, −2.40 to −1.140, respectively).

## Discussion

### Social Competence and Cognition in LPIs

LPIs enrolled in this study had low mean scores on both the S-M and GDDS scales. The clinical problems they experienced were low. They appeared to be normal and matured. However, their social cognition, competence, and motor skills were inferior when compared to FTIs of the same age. The gender, mode of delivery, maternal education, and financial status of the LPIs were the same as those of the FTIs. Still, this inferiority in social competence development existed. Previous studies indicated that LPIs were at an increased risk of social developmental problems (27). Children with a GA of 32–36 weeks had delayed social competence, which was reported at the age of 2 (23). Similar results were found in this study. Also, studies showed that LPIs aged about 30 months old showed delayed motor skills, language, adaption, and social skills. Compared with FTIs, LPIs had significantly lower scores in the five domains of the GDDS. However, the scores of LPIs were lower in only two subdomains of the S-M. The S-M did not show any difference between LPIs and FTIs in the socialization and communication subdomains. This may be due to the sample size or age. Therefore, social cognition is an important skill that helps to predict early inferior social skills in children. Our study also supported the idea that social cognition is a key variable of social competence (17).

In clinical practices, it was found that children with delayed motor abilities presented undesirable social skills. It is known that locomotive and self-help skills include abilities like walking, climbing stairs, eating, dressing, brushing teeth, etc. These are a child's early social skills and they are developed before the child is three. This was the main reason we wanted to identify not only low motor abilities but also poor locomotive and self-help skills in LPIs. Compared with FTIs, LPIs had lower mean scores in the occupation, communication, socialization, and self-regulation domains. However, there was no statistical significance. This trend may continue to become worse or become normal as the child grows older, which could be confirmed only if children were monitored in the long term. One study reported lower neuropsychological performance in LPIs at 3–4 years of age, and others presented these trends only at the age of five (28).

LPIs with inferior cognition presented low total or subdomain scores on the S-M scale. The correlation analysis showed that the domain functions in the GDDS had statistical correlations with the total score on the S-M. This indicated unfavorable cognitive influence. Gross motor skills and fine motor skills were positively correlated with the self-help and locomotive/locomotion skills, respectively. Our data indicated that the early signs of motor disorders correlated with inferior social development in children. Children with good motor and cognitive abilities will explore the outside world frequently and learn from it. This will help them acquire good social skills. Impaired social cognitive processing will lead to aggression, social anxiety, and low popularity with peers (29). Prematurely born neonates without major neurological deficits have a higher risk of facing difficulties in gross motor skills, social contact, and learning (30). Our findings showed the same. There were no statistical associations between motor skills (gross motor and fine motor) and the other subdomains of the S-M. Future study of these “no/weak” correlations will be conducted using a broader and larger population of LPIs and FTIs. A bigger sample size and longer study period are required to understand whether the “no/weak” correlations become stronger or diminish with age. Also, abilities such as communication skills, reading skills, participation in group activities, listening skills, the ability to take care of others, self-control, etc., must be observed to make a decision. Clinically compared with FTIs, LPIs had overprotecting and negative mother-infant dyads (e.g., the mothers were rated as controlling and the infants were rated as compulsive-compliant), which contributed to their poor performance on both the GDDS and S-M scales. It has been found that socio-biological vulnerabilities and cognition/motor deficits may increase the risk of socio-emotional behavioral problems (31). Thus, we should pay more attention to the development of both motor/cognition and social competence.

### Motor Disorders in LPIs

Two and nine LPIs were diagnosed with cerebral palsy and motor delay, respectively. Compared with FTIs, LPIs were more likely to have motor disorders (30). Neurophysiological hypotheses have been proposed to explain this. The following important developmental changes take place in the brain during late preterm gestation: A five-fold increase in white matter volume, increased myelination, neuronal connectivity, dendritic arborization, the formation of synaptic junctions, etc. (32). Therefore, even third-trimester preterm births will have limited fetal brain development (33). Compared with FTIs, “low risk” LPIs are thus more vulnerable to neurodevelopmental problems and hence require continuous developmental surveillance (28).

### Risk Factors for Social Development in LPIs

Physiological factors, motor disorders, and perinatal factors were found to influence the social development of LPIs. A multiple linear logistic regression analysis showed that GA, PIH, placenta previa, oligohydramnios, and motor disorders predicted a higher risk of delayed social skills. Therefore, it seems physiological immaturity exerts an unfavorable influence on LPIs' social development. Perinatal environmental risk factors associated with preterm birth may also increase the risk of delayed social development in LPIs and make them vulnerable to long-term socio-emotional problems (34). This study showed that motor skills were not only correlated with social competence but also predicted the risk to social development. LPIs with motor disorders and/or perinatal environmental risk factors were more likely to have problems in personal-social behavior, adaptability, self-help, and acceptance from peers. The complex interplay between these factors plays an important role in the development of future social and behavioral problems in LPIs.

### Limitations

This cross-sectional study aimed at studying the social development of LPIs up to the age of three. Therefore, children's behavior in school was not studied. Only if a longitudinal follow-up is conducted that explores the developmental disparity between LPIs and FTIs can there be a better understanding. Social competence should be evaluated using the Chinese Communicative Development Inventories, revised on the basis of Medical Care Development International. Cognitive functions such as working memory and executive functions were not studied. Therefore, the association between working memory, executive functions, and social competence remains unknown. Cognitive abilities in children can be studied in detail using some advanced tools like the Bayley Scales-III. The sample size was limited, and a few parents did not want to participate in the study. It is not clear if these refusals made a significant difference in the results of the study.

## Conclusions

In conclusion, we reported that LPIs are at greater risk of developing neurological problems than FTIs. It was observed that the LPIs had impairments in social competence and social cognition before 3 years of age. We found that cognition/motor abilities are associated with social competence and, along with certain perinatal factors, are predictive of inferior social adaptation.

Even LPIs with no major clinical problems after birth might develop some alterations in the future, so we recommend that regular monitoring of LPIs be carried out in the long term to prevent any developmental disorders from arising.

## Ethics Statement

This study was carried out in accordance with the recommendations of the Frontiers authors guidelines involving human subjects, the Ethics Committee of Xi'an Maternal and Child Health Care Hospital with written informed consent from all subjects. All subjects gave written informed consent in accordance with the Declaration of Helsinki. The protocol was approved by the Ethics Committee of Xi'an Maternal and Child Health Care Hospital.

## Author Contributions

JY conceptualized and designed the study, edited the questionnaires regarding the basic information about the subjects, identified children with developmental disorders, and drafted the initial manuscript. HY coordinated data collection and processing, and participated in interpretation of the results. MH participated in estimation of the Gesell Development Diagnosis Scale and the Normal Development of Social Skills from Infants to Junior High School Children scale for children. JZ checked the data and conducted the statistical analyses.

### Conflict of Interest Statement

The authors declare that the research was conducted in the absence of any commercial or financial relationships that could be construed as a potential conflict of interest.
